# From Zygote to Blastocyst—Molecular Aspects of Porcine Early Embryonic Development

**DOI:** 10.3390/cells15010015

**Published:** 2025-12-22

**Authors:** Beenu Moza Jalali, Marta Wasielak-Politowska

**Affiliations:** 1 Team of Reproductive Pathology and Translational Medicine, Institute of Animal Reproduction and Food Research, Polish Academy of Sciences, Trylinskiego 18, 10-683 Olsztyn, Poland; 2 Center of Gynecology, Endocrinology and Reproductive Medicine, Artemida, Jagiellońska 78A, 10-357 Olsztyn, Poland; m.wasielak.politowska@gmail.com

**Keywords:** porcine embryos, embryo genome activation, epigenetic reprogramming, early development

## Abstract

Early mammalian embryo development is a temporally regulated process initially governed by maternal factors during the first few cleavage divisions. In porcine embryos, the transition from oocyte to embryonic control occurs around the 4-cell stage. This developmental progression depends on embryonic genome activation (EGA), epigenetic reprogramming, metabolic cues, and extracellular signaling pathways. While fundamental aspects of early development are conserved across mammals, porcine embryos exhibit distinct molecular features, including unique EGA timing, altered regulatory gene expression, and a pronounced reliance on lipid metabolism. This review provides a comprehensive overview of recent advances in understanding the molecular mechanisms underlying early porcine embryo development, from fertilization to blastocyst formation. It summarizes molecular changes associated with the maternal regulation of initial embryonic divisions, genome activation, chromatin remodeling, and the role of transcription factors and metabolic pathways. Additionally, the review examines the impact of in vitro culture conditions on these molecular processes. A thorough understanding of these mechanisms is critical for optimizing embryo culture systems, improving developmental outcomes, and advancing agricultural biotechnology.

## 1. Introduction

Early embryonic development is regulated in a time-dependent manner, beginning with the transition from the fertilized egg to the implantation-competent blastocyst stage. This process involves critical events, including morphological and transcriptional changes driven by the interplay between cell positions and a series of molecular and epigenetic events that transform a single-celled totipotent zygote into a multicellular blastocyst. Embryonic cells follow divergent developmental pathways by expressing distinct gene sets, with a majority of differential gene expression regulated at the level of transcription initiation. Though morphological changes and general molecular mechanisms associated with early embryo development are highly conserved across mammals, a few interspecies differences exist, such as differences in the time spent at each stage and the timing of cell lineage commitment [1]. After fertilization, development depends on maternal gene products stored in the oocytes until the transcription machinery is activated in the embryo. The time period of embryonic development under maternal control, during which there is almost no transcriptional activity of its own, varies across species [2]. The products of maternal genes, called maternal-effect genes (MEGs), controls the morphogenesis of the fertilized egg. In some cases, such as in mice, their presence persists even after MEGs are expected to have degraded [3,4]. Further embryo development includes essential stages, such as zygotic or embryonic genome activation (EGA), progression to the morula stage, lineage specification, and blastocyst formation [5,6]. Understanding these developmental transitions at the molecular level is essential for improving reproductive efficiency and for determining the factors that affect developmental competence. The pig is a valuable model for studying early mammalian development, and its physiological and genomic similarities to humans make it significant in developmental research and genetic engineering [7,8]. Despite improvements in assisted reproductive technologies and in vitro culture systems, porcine in vitro-produced (IVP) embryos still develop less efficiently than their in vivo counterparts and those of rodent or cattle embryos [9]. This low efficiency arises from factors such as polyspermy, suboptimal culture conditions, and higher lipid content in porcine embryos that can increase oxidative stress through lipid peroxidation [10,11]. Recent high-throughput transcriptomic studies have broadened our understanding of early porcine embryo development. These studies using in vivo and IVP embryos have reported the expression of many genes that coordinate the degradation of maternal RNAs, initiate EGA, control cell divisions and chromatin remodeling, regulate energy metabolism, and influence cell fate decisions [12,13]. Additionally, comparative studies between in vivo-derived, IVP, parthenogenetically activated (PA), and somatic cell nuclear transfer (SCNT) embryos have provided important insights into the molecular changes associated with maternal-to-zygotic transition and EGA, changes in epigenetic reprogramming, including differences in DNA methylation, histone modifications, and gene expression that drive proper embryo development [14,15,16]. This review combines current knowledge about the molecular control of early embryogenesis in pigs, from fertilization to blastocyst formation. It emphasizes transcriptional and epigenetic control mechanisms and metabolic regulations of embryo development. Moreover, we address recent insights into the dysregulation of genes involved in metabolism, oxidative stress, and epigenetic remodeling under in vitro conditions. Understanding these mechanisms is crucial for refining porcine IVP systems and improving embryo quality.

## 2. Maternal-Effect Genes Controlling Early Developmental Events

### 2.1. Maternal Regulation

Oocyte quality is crucial for successful embryo development. During the period of transcriptional silence in the zygote, oocyte-derived factors guide early embryo development. The significance of these maternal contributions was demonstrated by SCNT experiments, which showed that an oocyte can reprogram a differentiated cell into a totipotent zygote [17]. This occurs because the oocyte is loaded with maternal factors such as macromolecules, ribosomes, and cytoplasmic organelles needed for post-fertilization development during transcriptional silence. Many genes, known as MEGs, are transcribed in oocytes but are not immediately translated, as they are responsible for early embryo development. Maternal transcripts are essential for oocyte nuclear maturation, proper processing of the sperm nucleus, organization of the two pronuclei, initial embryo divisions, embryonic genome activation, and the first cellular differentiation events [18,19]. Embryonic genome activation is a key stage during which the embryo begins to rely on mRNAs and proteins produced through new transcription from its own genome [20]. During oocyte cytoplasmic maturation, maternal transcripts are stored and regulated by polyA tail length, which protects them from translation. After meiosis resumption, these transcripts are polyadenylated and translated [21,22]. From a transcriptomic perspective, the onset of EGA is marked by increased use of transcripts required for genome activation, along with the simultaneous degradation of transcripts that could otherwise cause premature activation [23]. In mammals, knocking out maternal-effect genes prevents embryonic development after 2–3 cleavages [24,25,26]. These proteins are involved in various aspects of early development, including maternal mRNA degradation, epigenetic reprogramming, signal transduction, protein translation, and initiation of embryonic genome activation [2].

### 2.2. Maternal-Effect Genes and Their Degradation

A few maternal-effect genes have been identified and characterized in pig oocytes and early embryos. Their expression is important for embryonic development, as dysregulation of their expression leads to defects in embryogenesis. Calcium signaling plays an important role in oocyte maturation and early embryo development by controlling the dynamic expression of maternal genes [27]. The MEGs identified in porcine embryo include *cyclin B1 (CCNB1)*, *NLRP5*, *ZAR-1*, *DDB1*, *DDB3*, *SEBOX*, *YAP-1*, *SIN3A*, and *NPM2* [19,28]. *NLRP5*, also known as *MATER*, and *ZAR-1* are two of the earliest known MEGs identified in mouse oocytes required for early embryo development [25,26]. *MATER* and *ZAR-1* are exclusively expressed in porcine ovaries [28,29] and not in the other reproductive parts. In pigs, MEGs are abundantly expressed in mature oocytes and early cleavage-stage embryos. Still, their expression declines dramatically after the 4-cell stage, and remains low with the exception of *DDB1*, which is also expressed at the morula and blastocyst stage [28,29,30]. Inhibition of any of the genes among *SEBOX*, *DDB1*, and *YAP* in oocytes or *NLRP5* in zygotes by RNA interference inhibits developmental progression. Whereas *NLRP5* maintains early cleavage divisions, *DDB1* is important for the expression of lineage and pluripotency-related genes *TEAD4*, *CDX2*, *OCT4*, *NANOG*, and *SOX2* [29,30]. *SEBOX* is essential for blastocyst formation and facilitates the selective degradation of maternal RNAs [31], while YAP supports trophectoderm formation and epithelial barrier function through the transcriptional regulation of genes responsible for the formation of the epithelial junction and lineage specification [32].

Components of cell-cycle regulation, such as *CCNB1* and *SIN3A*, are maternally provided [33,34]. The expression pattern of SIN3A, a scaffold protein, is species-specific with a minimum at EGA in pigs, mice, and humans, indicating its maternal origin [34]. Its silencing causes developmental arrest at the 2-cell stage of porcine embryos, and its role in development is suggested to be through the regulation of CCNB1 expression. The transcriptional control from oocyte to embryo occurs in a tightly regulated and time-dependent manner. This process, maternal-to-zygote transition, is associated with the timely degradation of MEGs and reprogramming of chromatin for the EGA [35,36]. The degradation of maternal factors is largely species-specific. A recent study compared the expression of six different maternal origin genes and proteins in mice, bovine, and porcine oocytes and embryos during the EGA window. Among six proteins, protein inhibitor of activated STAT4 (PIASY) was detected in oocytes and early embryos and displayed a decrease in abundance that coincided with the major wave of EGA in each species, suggesting its conserved role despite differences in the timing of EGA between mice (early 2-cell), pigs (4-cell), and cattle (8-cell) [37]. The stability of MEGs is regulated by processes, such as mRNA adenylation/deadenylation, and the expression of transcription factors, such as YAP1-TEAD4 in pigs and humans [38,39]. The exact mechanisms by which maternal transcripts are degraded in porcine embryos are not known; however, the expression of the hippo-signaling family, such as *YAP1* and *LATS2*, is found to be at its peak in matured oocytes and 1-cell stage embryos [40]. The silencing of these genes impaired embryo development and expression of genes involved in trophectoderm and inner cell mass lineage specification. Future research should investigate whether these transcription factors play a role in maternal transcript clearance in pigs. Although maternal protein degradation in porcine embryos, as in other species, was suggested to rely on the ubiquitin–proteasome system [41], autophagy might be another mechanism supporting the process [42]. One-cell cleavage stage embryos have the highest autophagic activity and expression of autophagy-related genes, *ATG5*, *Beclin1*, and *LC3* [42,43]. The silencing of *LC3* in porcine parthenotes, a central player in autophagy, upregulates the maternal transcripts, such as *CCNB1*, *c-mos*, *DPPA3*, and *GDF9*, suggesting its role in maternal transcript degradation [43]. These findings establish that the degradation of maternal mRNAs and proteins in pigs is not a passive process but a precisely orchestrated molecular transition directed by maternal-effect genes that prepares the embryo to take control of development.

## 3. Early Embryo Development and Genome Activation

Fertilization triggers Ca^2+^ release in the egg, leading to the degradation of CCNB1 and the subsequent release from meiotic arrest, allowing anaphase initiation. Post-fertilization development starts with mitotic cleavage divisions of the zygote, which requires the inactivation of maturation/M-phase promoting factor (MPF), allowing for the separation of the sister chromatids [44]. In addition, MAPK inactivation is also essential for pronuclear formation after fertilization or parthenogenetic activation [45]. The initial cell division in the embryo lacks gap phases as the S-phase is directly followed by mitosis (M-phase) [46]. However, in in vitro-derived embryos, the long gap phase is observed only after the second cell division, which might be a reason for the long duration of the 4-cell (~50 h) stage in an in vitro-developing porcine embryo [47]. Cell divisions after fertilization lead to increased cell numbers with no significant growth, resulting in the formation of a blastocyst. Initially, the cell divisions are controlled by maternally derived CCNB1, CDC25C, and p34cdc2 (cyclin-dependent kinase, CDK1) among others [48]. A decline in the expression of CCNB1 and CDC25C occurs till the 4-cell stage, which coincides with the EGA [49,50]. Cyclin B1 is essential for early development in pigs; its inhibition results in developmental arrest at the 2-cell stage [34].

Timely and precise cell division and DNA replication are important for embryonic fate and maintaining genome integrity [51,52,53]. The cell cycle is controlled by oscillating expression and the activity of the cyclin superfamily and cyclin-dependent kinase (CDKs). CCNB1 mRNA is present in a polyadenylated form at significantly higher levels in MII oocytes and the 2-cell embryos, followed by a significant decrease till the 4-cell stage [48]. On the other hand, *CDC2* is deadenylated in metaphase II oocytes and early embryos [54]. Whereas *CCNB1* decreases over the 4-cell stage, there are no significant changes in protein levels [49]. It was suggested that CCNB1 can be post-transcriptionally regulated during the initial stages of embryo development [50]. Though CCNB1 levels positively correlate with the developmental competence of porcine early embryos, similar to humans [55], it is likely not the only protein regulating the cleavage of porcine embryos to the 8-cell stage. Other cell cycle-related genes, such as *CCND3*, *CDKN2*, and *TFDP1*, can play a role in the cell cycle during cleavage in the early embryo [53]. Their dysregulation results in a slower cleavage rate. Cyclin-dependent kinase 2 (CDK2), a G1/S cell-cycle checkpoint kinase, is important for cell-cycle regulation and early cleavage of porcine embryos. Though CDK2 is dispensable for cell-cycle progression, disruption of its activity results in delayed cleavage and induces developmental arrest in porcine parthenogenetic (PA) or in vitro-derived embryos before they can reach the blastocyst stage [56]. Cell-cycle arrest in early embryos can be caused by chromosomal aberrations or the accumulation of DNA damage. CDK2 may participate in early embryo cleavage by acting as a DNA damage sensor.

### Embryonic Genome Activation

**Figure 1:**
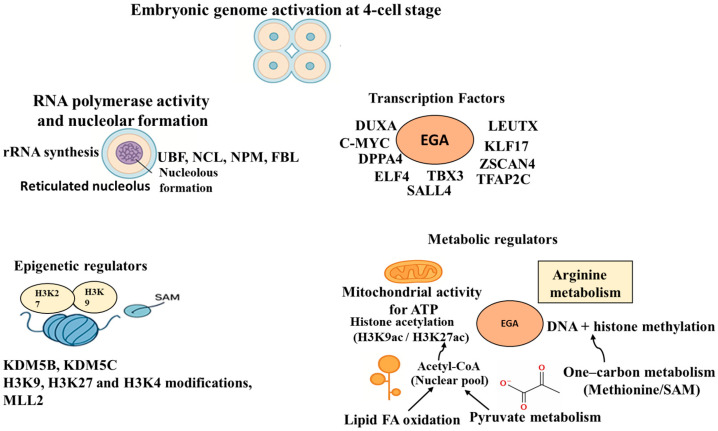
Major players associated with porcine embryonic genome activation at the 4-cell stage.

Embryonic genome activation represents a critical developmental milestone [57] and is associated with many molecular changes summarized in Figure 1. Both, in the case of domestic animal and human embryos, the developmental block observed during in vitro culture is the result of perturbation in EGA and is the main cause of the embryo’s failure to reach the blastocyst stage [20,58].

In the porcine embryo, nucleoli are absent at the pronuclear and 2-cell stage [47,59,60]. Embryonic transcription appears to initiate at the 4-cell stage, evidenced by changes in nucleolar structure, such as the transformation of nuclear precursor bodies (NPBs) into a functional reticulated nucleolus, alterations in the nuclear matrix, beginning of rRNA synthesis [61,62], along with the activation of RNA polymerase transcription [63]. NPBs transport nucleolar components from the oocyte to the embryo and are also suggested to participate in genome remodeling [64]. Nucleolus formation marks the transcription of ribosomal RNA genes [62,65]. The maternally inherited rRNA partially initiates nucleolar assembly, but for complete nucleolar formation, de novo protein synthesis by the embryo is essential [62,63]. RNA polymerase I is expressed after embryonic gene transcription commences. This corresponds to the appearance of many nucleolar proteins associated with the rRNA transcription, processing, and ribosome assembly, such as upstream binding factor, topoisomerase I, fibrillarin, nucleolin, and nucleophosmin [59]. Among these proteins, nucleophosmin and nucleolin are possibly of maternal origin [63]. Embryos where RNA polymerase activity is inhibited do not develop to the blastocyst stage [66]. Across multiple independent transcriptomic studies, the consensus pattern in pig embryos is that a major EGA occurs around the 4-cell stage. Single-cell RNA-seq profiling of individual blastomeres [67] and several bulk comparative studies document the sharpest transcriptomic remodeling between the 2-cell and 4-cell stages, while the 2-cell stage shows a minor wave of EGA, and the 4-cell embryo shows the largest number of upregulated zygotic transcripts and concurrent maternal RNA clearance [14,67]. The EGA in pigs at the 4-cell stage is observed in in vitro-produced (IVP), parthenogenetically activated (PA), and many in vivo comparisons [12,13,68,69]. However, SCNT embryos show delayed EGA [70]. Transcriptomic studies have suggested several genes as possible regulators of EGA in pigs, including *ELF1A*, *DPPA2*, *ZSCNA4*, *SIRT1*, *DNMT1*, *KLF17*, *USP26*, and *YTHDC2* [16,67,70,71].

Transcription factors (TFs) play a pivotal role in activating the transcriptional machinery and, along with chromatin remodeling, initiate the EGA programs. Some of these TFs, called pioneer TFs, such as DUX and LEUTX, can bind compact nucleosomal DNA, enabling chromatin remodeling and gene expression. Members of the DUX family are transiently expressed prior to EGA in mice (Dux) and humans (DUX4) and induce the expression of EGA genes [72,73]. A recent study has highlighted the role of DUXA, a member of the DUX family, in the EGA of porcine SCNT embryos. Its knockdown affects the expression of EGA-related genes, *TDG*, *SNAI1*, *RSRP1*, *TFAP2C*, *ZSCAN4*, *LEUTX*, and *KLF17*, and early development in porcine embryos [74]. A significant advance to the role of TFs in EGA comes from Zhou et al. [36], who identified LEUTX, a PRD-like homeobox transcription factor, as a key activator of human and porcine embryonic genome activation. In pig embryos, LEUTX is transiently expressed in 4-cell in vivo-derived embryos during the EGA window. Interestingly, its overexpression in SCNT embryos restores EGA failure and their cloning efficiency. Transcriptomic profiling of porcine in vivo and SCNT embryos identified many TFs during the EGA. The activated TFs included *c-MYC*, *KLF4*, *EED*, *RBMX*, *CRABP2*, *ATF3*, and *TGIF1*, and their reduced expression in SCNT embryos correlated with their impaired EGA [70]. Among TFs, the perturbation of c-MYC function using the small molecule inhibitor, 10,058-F4, inhibited EGA gene expression and increased apoptosis in blastocysts, demonstrating that c-MYC is associated with robust EGA and early embryo viability. Transcription factor, P-TEFb, and RNA polymerase II (Pol II), drive gene expression by facilitating transcriptional elongation. In pigs, p-TEFb, through its catalytic subunit CK9, is suggested to play a role in Pol I and Pol II-dependent transcription and in EGA [75]. A study by Du and coworkers suggests *SALL4*, *NANOG*, *KLF4*, and *KLF17* as TFs associated with EGA in porcine IVP and PA embryos [67]. Complementary evidence suggests *ELF4* and *TBX3* are other essential regulators of EGA and early embryo development. These TFs maintain genome integrity by reducing DNA damage and regulating dynamic changes in histone and DNA methylation and histone acetylation [76,77].

Metabolic regulation is increasingly recognized as a key driver of EGA, and the role of mitochondrial metabolic enzymes is conserved in mammalian EGA. The major wave of EGA in porcine embryos coincides with a marked increase in mitochondrial activity and oxidative phosphorylation, which provide the energy required for chromatin remodeling and the transcriptional activation of early embryonic genes [67,70]. Consistent with this observation, inhibition of mitochondrial function via stress conditions leads to reduced expression of EGA markers and developmental arrest in 4-cell stage porcine embryos, indicating a direct requirement for energy metabolism in this process [78]. Pyruvate metabolism plays a complementary role in EGA in porcine PA embryos by generating nuclear acetyl-CoA, which maintains histone acetylation at H3K9 and H3K27 [79], thereby promoting transcriptional activation during EGA. Amino acid-dependent pathways are also crucial for EGA. Arginine (Arg) metabolism-related genes are activated in in vivo-derived embryos during this period. Whereas the removal of Arg from the in vitro culture medium of embryos causes 4-cell arrest and a decrease in EGA genes, its supplementation reverses these effects [71]. One-carbon metabolism, through the synthesis of S-adenosylmethionine (SAM), is essential for establishing appropriate DNA and histone methylation patterns. Interfering with methionine metabolism through the silencing of methionine adenosyltransferase 2A (MAT2A), an enzyme that generates SAM, impairs histone methylation and EGA in porcine PA embryos [80]. It has been well established that the genes involved in the TCA cycle, glycolysis, amino acid metabolism, and fatty acid oxidation are dynamically regulated at the 4-cell stage, and embryos derived from SCNT often display dysregulation of these pathways, correlating with impaired EGA [70]. Moreover, the expression of genes involved in lipid metabolism shows significant alterations during the maternal-to-zygotic transition. These changes correlate with the expression of EGA-related genes, suggesting lipid metabolism as an indicator of EGA in pigs [81]. A high content of fatty acids in porcine embryos sustains energy needs by providing acetyl-CoA for ATP generation. Fatty acid oxidation is linked to histone acetylation through nuclear localization of acetyl co-enzyme A synthetases ACSS1 and ACSS2, affecting EGA in pigs [82].

## 4. Epigenetic Modifications During EGA

In addition to transcriptional activation, the embryonic genome undergoes extensive epigenetic remodeling [83], including DNA methylation, histone modifications, chromatin accessibility, and 3D chromatin organization after fertilization to restore zygotic totipotency and enable embryonic development. The timing of epigenetic modifications, such as histone modifications that regulate chromatin structure and affect gene expression, coincides with EGA. Though it is not well understood whether EGA leads to epigenetic modifications or whether these modifications drive EGA, in zebrafish, epigenetic modifications related to the expression of developmental genes were observed before EGA [84]. The methylation of histone H3 lysine residues, such as lysines 4 and 27, is an epigenetic mark that can lead to transcriptional activation or repression, respectively. Transcriptional activity also depends upon the mon-di- or tri-methylation of lysine residues. During cleavage stages of development, global H3K27 methylation patterns undergo a dramatic change [85,86]. In pigs, whereas mono-methylated H3K27 is present in the nucleus of oocytes and throughout the early embryo development till the blastocyst stage, levels of tri-methylated H3K27 are highest in oocytes [85]. Previous reports on H3K27 trimethylation in cleavage stage embryos are inconsistent. While some studies detected H3K27me3 in only one pronucleus [85,87], Gao et al. report its presence in both pronuclei [88]. After cleavage, a decrease in H3K27me3 was observed in 2- and 4-cell embryos [85,88]; in contrast, Marinoho et al. report no measurable decline at these stages [87]. These observations in pigs are in contrast to murine embryos, where H3K27me3 is detected in maternally derived chromatin and in blastomeres from 4-cell embryos and blastocysts [89]. On the other hand, H3K27 acetylation is generally maintained throughout porcine development [87]. H3K27 methylation is shown to be catalyzed by the histone methyltransferase EZH2 in porcine PA embryos [90].

The work by Glanzner and co-workers elucidated the importance of histone modification in porcine EGA. Changes in H3K4 and H3K9 methylation, as well as in the expression of lysine demethylases during the EGA period, were associated with the developmental competence of early embryos [91,92]. Histone demethylases KDM5B and KDM5C are highly expressed around EGA, and blocking their expression impairs porcine embryo development. The attenuation of histone demethylase function delays EGA by impairing RNA transcription in embryos and correlates with the downregulation of EGA markers, such as EIF1AX and EIF2A. Histone demethylases have long-term consequences for cell differentiation and development by regulating EGA, DNA damage repair, and the expression of pluripotency genes [92,93]. The expression of epigenetic-modifying enzymes, such as KDM5B, is under the control of transcription factor AP-2 gamma, and its silencing in porcine oocytes interferes with embryonic development, highlighting the importance of histone methylation modifications in embryo growth [94].

The involvement of epigenetic mechanisms, especially histone methylation and demethylation, was further emphasized by oocyte-specific sharp H3K4me3 peaks at the promoters of EGA-related genes. These sharp peaks transitioned to broader domains after fertilization and became sharp again during EGA [95]. The transitions between broad histone H3K4me3 domains to sharp peaks regulate EGA during early embryo development in mice and humans [96,97]. The maintenance of a broad H3K4me3 pattern represses gene activity during cleavage-stage embryo development in pigs and was suggested to control the entry of embryos to the EGA stage [95]. This is in contrast to mice, where pre-EGA transcription silence is not dependent on broad H3K4me3 domains [98]. The removal of H3K4me3 demethylases, such as KDM5B and KDM5C, maintains the broad H3K4me3 and interferes with the expression of EGA genes in pigs, mice, and cattle [95,96,99]. In another study, the expression of MLL2, an H3K4 trimethyl transferase, was higher after fertilization and was significantly downregulated after the 4-cell stage of porcine embryo development [100]. Its role in initiating the transition from a broad domain to a sharp peak before EGA needs to be investigated. Interestingly, Bu and co-workers observed the establishment of H3K4me3/H3K27me3 bivalency at the promoters of EGA genes during the morula stage of embryo development, suggesting the possibility that bivalency may serve to repress EGA gene expression, leading to an exit from totipotency and EGA [95]. In addition to histone methylations, which are covalent modifications, non-covalent changes in chromatin structure and 3D chromatin organization also govern transcription activation or repression. These processes are well-established in cell differentiation and development [101]. Changes in chromatin structure are also brought about by the non-covalent modifications by ATP-dependent chromatin remodeling complexes (CRCs), such as switch/sucrose non-fermentable (SWI/SNF), imitation switch, inositol-requiring 80, and chromodomain-helicase DNA-binding protein complexes. The CRCs, such as SWI/SNF, non-covalently move or alter the nucleosome position by interacting with DNA and histones via specific domains. SWI/SNF complexes are assembled from many components and sub-units that are shown to regulate EGA in murine embryos [102]. The role of AT-rich interactive domain-containing protein 1A (ARID1A), a subunit of the SWI/SNF complex, was established in porcine oocytes and embryos [103]. It is expressed highly in germinal vesicle-stage oocytes and in blastocysts, with a decreased expression at the 4-cell stage. It is suggested to be required for the proper cleavage of embryos up to the 4-cell stage, as its silencing leads to impaired cleavage, potentially interfering with the EGA, and decreased developmental competence [103].

Over the last 15–20 years, owing to techniques such as high-throughput chromosome conformation capture (Hi-C), studies have now reported the importance of changes in the 3D reorganization of chromatin during early embryonic development [104]. The hierarchical packaging of chromatin domains in the nucleus of eukaryotic cells is essential for gene transcription and repression [105]. Whereas topologically associated domain (TAD) establishment consistently coincides with EGA in pigs and many other species [106,107,108], in mice, TADs appear after zygotic genome activation [109]. On the other hand, chromatin superdomains are more prevalent in porcine embryos. As lipids, such as cholesterol, can cause chromatin compaction [110], it was suggested that the superdomains during porcine embryo development are possibly due to the high cytoplasmic and nuclear lipid content of porcine oocytes [111].

The rapid development of high-throughput sequencing technologies has shed light on the importance of long non-coding RNAs (lncRNAs) in mammalian embryonic development [112]. Recently, the role of long non-coding RNA (lncRNA) in porcine EGA has been reported [113]. The expression of lncRNAs in porcine in vivo embryos coincides with the EGA, as their expression was noted at the 4-cell stage and in blastocysts. Additionally, lncRNA LOC102165808, termed lncT, supports in vitro embryo development by modulating histone and DNA methylation and the expression of SIN3A, a downstream target of lncT [113]. Another lncRNA, lncFKBPL, is expressed in 4-cell stage PA embryos and morulae. It is suggested to facilitate EGA indirectly through CDK9 stabilization and increasing Pol II phosphorylation levels [114].

## 5. Embryo Metabolism

### 5.1. Carbohydrate Metabolism and Early Embryo Development

The role of metabolism in generating energy for preimplantation embryo development has been known for a long time [115,116]. Metabolism has been studied mainly by identifying the conditions that allow for the in vitro development of the embryo. A recent review analyzed in depth the effect of culture conditions on metabolism and developmental competence of IVP embryos [117]. It is now well known that metabolism influences not only energy production but produces various metabolites that are utilized for gene expression, cell signaling, alterations in chromatin and DNA states, and cell fate decisions [118]. Energy requirements change during early development with marked differences between cleavage-stage embryos and blastocysts. During the initial cell divisions, embryo metabolism is slow, and development is supported primarily by the maternal transcripts and proteins stored during oocyte maturation [2]. Studies report that a very high or very low metabolism in mammalian species is associated with developmentally compromised embryos [119,120,121]. The metabolic pathways active during early development are species-specific [122], and early embryos use pyruvate, lactate, carbohydrates, and amino acid metabolism as an energy source. The transition from the 2-cell to 4-cell stage of early in vivo embryo development is associated with a significant increase in gene transcription, and a large number of these are related to metabolic pathways and are important for EGA [12,71]. Developing embryos exhibit significant metabolic plasticity, as their metabolism can adapt in response to various factors and developmental stages. This adaptability is crucial for supporting the diverse energy needs and cellular processes required for proper development [123]. Pyruvate and lactate are the main sources of energy during initial development, followed by an increase in glucose metabolism during blastocyst formation. In the absence of pyruvate, mouse embryos cannot grow beyond the 2-cell stage [124], and bovine embryos cannot grow beyond the morula stage [125]. However, it was observed that porcine embryos increase reliance on pyruvate at the blastocyst stage and utilize glucose as the primary source of energy [126]. In earlier studies, the use of glucose was shown to be beneficial to the in vitro development of porcine embryos, and the addition of glutamine to media containing glucose yielded better results [126,127,128,129,130,131]. Metabolism measurements showed that, whereas in vivo-derived porcine embryos use glucose preferentially through glycolysis during the cleavage stage and pre-implantation development, in vitro-derived glycolysis is preferred after the 2-cell stage [126]. Pig embryos are known to metabolize glucose through the pentose phosphorylation pathway (PPP) to provide NADPH for biosynthesis. The pentose pathway activity is higher till the 8-cell stage of embryo development, followed by glucose metabolism by glycolysis [127]. In addition to glycolysis, the metabolism of glucose through the TCA cycle is also active in porcine embryos, albeit at lower levels during the cleavage stage, with a significant increase in TCA activity as the development reaches the morula and blastocyst stages.

However, there is evidence that glucose in in vitro embryo culture media during initial cleavage divisions can cause oxidation stress [127] and a difficulty in overcoming the 4-cell block [132]. Consistent with these observations, in vitro porcine embryo development, in terms of blastocyst formation and total number of cells in a blastocyst, is benefited by the presence of pyruvate and lactate in the culture media during the initial cleavage divisions up to day 2 of the culture post-in vitro fertilization, followed by the addition of glucose [133,134]. The benefit of glucose in in vitro culture media after the 4-cell stage coincides with the EGA [132]. It is possible that after EGA, an increase in the expression of oxidation stress response genes can alleviate the increase in ROS caused by glucose metabolism through NRF2-mediated response [13]. The demands for carbohydrates in in vivo-derived embryos are initially low, followed by an increase at the blastocyst stage [1,135].

### 5.2. Fatty Acid Metabolism and Early Embryo Development

Porcine embryos have large stores of endogenous lipids, mainly stored as triglycerides in the form of lipid droplets whose density changes during early embryo development. In addition to glucose, lipids serve as an important source of intracellular energy storage through mitochondrial β-oxidation of fatty acids, a process that produces more ATP than the oxidation of glucose [136]. Fatty acid metabolism is important for oocyte maturation and early embryo development [137]. The inhibition of fatty acid metabolism, when glucose is absent from the in vitro culture media, impairs embryo development [130]. Lipid droplets in a porcine zygote are composed of unsaturated and saturated lipids, with high levels of unsaturated hydrophobic lipids, in addition to esters, free fatty acids, and phospholipids [138]. The authors observed that total lipid content during the development of the zygote-to-morula stage remains unchanged, then decreases during the blastocyst stage [138], which is accompanied by a corresponding increase in oxygen consumption [136]. The decrease in lipid content in the blastocyst is suggested to result from increased β-oxidation, consistent with high oxygen consumption to support the energy demands during this stage [139,140]. However, the expression of genes associated with lipid metabolism show an increasing trend from the 1-cell stage to the blastocyst. The expression of enzymes responsible for lipid metabolism via mitochondrial beta-oxidation, is enriched in porcine blastocysts [81]. Recently, the crucial role of unsaturated lipid-mediated plasma membrane fluidity in the regulation of mouse and human embryo development was highlighted [141]. These results in mice and humans are also supported by observations in pigs, where during the maternal-to-zygotic transition, embryos express higher levels of genes associated with fatty acid metabolism and storage, such as stearoyl-CoA desaturase 1 (SCD1) and PPARγ. Whereas SCD1 converts saturated fatty acids into monounsaturated fatty acids and regulates fatty acid composition, PPARγ regulates fatty acid storage. The inhibition of Stearoyl-CoA desaturase 1 during the cleavage stage interferes with embryo development. These embryos have fewer and smaller lipid droplets and decreased morula compaction [142]. It is evident from many reports that saturated fatty acids, such as palmitic acid, inhibit oocyte maturation and subsequent embryo development [143,144], whereas unsaturated fatty acids, such as oleic acid, docosaexaenoic acid, and eicosapentaenoic acid, have beneficial effects on these processes during in vitro maturation and in vitro culture of embryos [145,146].

Lipid droplets interact with the mitochondria to create energy-efficient metabolic units [147]. This interaction is facilitated by small molecules, such as L-carnitine, that regulate the transfer of fatty acids to mitochondria for β-oxidation [148]. L-carnitine has a positive effect on embryo development by facilitating the efficient utilization of lipids [149]. In fact, L-carnitine, at a concentration up to 12 mM, can sustain in vitro culture in the absence of pyruvate, lactate, and glucose, pointing towards the importance of lipid metabolism for energy needs during embryo development [149].

In early embryos, although oxidative phosphorylation is functional, they use fatty acids for energy. A study comparing genes associated with fatty acid biosynthesis and elongation in pigs and mice suggested that lipid use for energy storage is unique to pigs [150]. However, recent studies have shown the importance of lipid metabolism in embryo development across the species [147]. Porcine embryos utilize lipids as an energy source in the absence of pyruvate, and though porcine embryos develop with lipid alone as a substrate, they can also oxidize pyruvate as an energy source, reflecting the plasticity in the embryo metabolism [123,149].

### 5.3. Amino Acid Metabolism and Embryo Development

Amino acids, depending on their size and type (zwitterionic, acidic, or basic), are transported across membranes via specific transporters. In pigs, amino acids such as L-alanine and L-leucine are transported by a Na^+^ dependent B^0,+^ system [151] in oocytes and in embryos up to the 4-cell stage. It is possible that, before EGA, the early embryo might depend on oocyte-specific amino acid transporters. Amino-acid transporters are developmentally regulated. In porcine embryos, L-aspartate and L-glutamate transport through the Na^+^-dependent X-AG system (carrier of acidic amino acids) increases during the blastocyst stage, but unlike in mice, it is absent in the oocytes [152]. In contrast, the activity of Na^+^-dependent transport system B^0,+^ that carries zwitterionic amino acids, such as alanine and leucine, and cationic amino acids, is high only in porcine oocytes [151] and is lost during the development of the embryo. The B^0,+^ amino acid transport system is active in species with invasive placentation, where it induces trophoblast motility and penetration by specifically activating mTORC1 signaling [153,154]. The amino acids, leucine and alanine, are transported by the Na^+^-independent system L in early porcine blastocysts [151]. The primary role of system L is to regulate intracellular amino acid concentrations for protein synthesis [155]. Thus, the requirement for amino acids is stage-specific in early embryo development.

Amino acids are one of the important constituents of the oviduct and uterine fluid [156,157]. Their transporters, metabolism, and metabolites play essential roles in embryo development through processes, such as osmoregulation, nucleotide biosynthesis via one-carbon metabolism, and epigenetic regulation [153,157,158]. Similar to many other species, porcine oviduct and uterine fluid contain many amino acids, with glycine and alanine being present at the highest concentrations and taurine and hypotaurine at lower concentrations [159,160]. Both essential and nonessential amino acid metabolism is reported to support early embryo development.

The importance of amino acids in embryo growth was initially evaluated by adding individual or combinations of amino acids and monitoring their appearance or depletion in the culture media [161]. The amino acids reported to have a positive effect on embryonic growth include arginine, glutamine, and glycine, among others. Glycine is present at the highest concentrations in the oviduct and uterine fluid of sows [162]. Glycine metabolism, or the glycine cleavage system, catabolizes glycine to carbon dioxide, ammonia, and a one-carbon unit essential for the synthesis of DNA and nucleic acids. This system is active in porcine oviduct cells and in embryos, potentially contributing to embryo development [163]. Glycine supplementation in in vitro maturation and in vitro culture medium supports oocyte maturation and the developmental potential of PA and IVP embryos by modulating oxidation stress and apoptosis [164] and by increasing mitochondrial membrane potential, regulating oxidative stress, and blocking lipid peroxidation [165].

Arginine metabolism-related genes such as *ODC1*, *GLUD1*, *OAT*, and *ASS1* are expressed at higher levels in 4-cell in vivo-derived embryos compared to earlier stages or morula. Their expression is highly correlated with the EGA genes, such as EIF1A. The absence of arginine in the in vitro culture media causes a 4-cell block, as the embryo cannot progress to a blastocyst [71]. On the other hand, the supplementation of in vitro culture medium with arginine aids embryo development by inducing glycolysis through the increased expression of *HK1* and *HK2* and by inhibiting pyruvate dehydrogenase kinase 1, an enzyme that blocks the TCA cycle [166].

In pigs, patterns of amino-acid turnover in culture medium are stage-dependent and correlate with embryo viability and changes in amino acid transporter expression. Whereas methionine uptake by cleavage-stage embryos decreases drastically after the 2-cell stage, arginine and glutamine are taken up by expanded and early blastocysts, respectively [167,168]. The increased glutamine use by early blastocysts also coincides with increased glucose metabolism, and the intermediates produced by glycolysis and glutaminolysis can be used for biosynthesis. The significance of glutamine in porcine early embryo development is highlighted by the observation that only glutamine in the culture medium can support the growth of in vivo-produced cleavage-stage embryos to the blastocyst stage [128]. It acts as an energy source and can replace glucose for the TCA cycle, supporting early embryonic development [169]. Glutamine alleviates oxidative stress and DNA damage [170]. It was also reported that expanded blastocysts consume significantly less glutamine than arrested porcine blastocysts, as the latter may require higher glutamine levels to alleviate oxidative stress [168]. Consistent with this study, glutamine has recently been proposed as a non-invasive biomarker of human embryo quality, as poor-quality aneuploid embryos consumed significantly more glutamine than good-quality embryos [171]. In addition, glutamine acts as a metabolic regulator, promoting mitochondrial activity and mTOR signaling pathways, which are crucial for cell proliferation [172].

**Figure 2:**
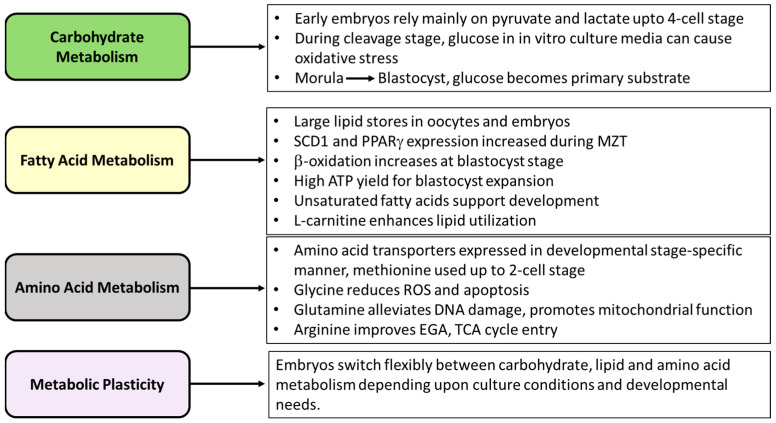
Metabolic control of early porcine development. MZT—maternal-to-zygotic transition.

Recently, a porcine trophectoderm cell model identified potential nutrients that promote embryo development and implantation. A combination of lysine (1.87 mmol/L), methionine (0.82 mmol/L), tryptophan (0.23 mmol/L), and arginine (3 mmol/L), with the ratio of 1:0.43:0.12:1.60, increased the expression of key genes involved in early embryonic development [173]. The stage-specific use of metabolic substrates is summarized in Figure 2.

## 6. DNA Damage and Repair in Early Porcine Embryos

Early developing embryos face exposure to both internal stressors, such as reactive oxygen species, and external stressors, including ionizing radiation and genotoxic chemicals [174,175]. These exposures can induce DNA damage, particularly double-stranded breaks (DSBs), which pose a significant threat to genomic stability [176]. If unrepaired, DSBs can disrupt embryo development. To counteract DSBs, embryos activate a DNA damage response (DDR), employing mechanisms such as homologous recombination (HR) and non-homologous end joining (NHEJ). These repair pathways are regulated by kinases, including ataxia telangiectasia-mutated (ATM), ATM-and-RAD3-related (ATR), and DNA-dependent protein kinase [177]. In in vitro- and SCNT-developed porcine embryos, DSBs are indicated by the phosphorylated form of histone 2X (H2AX) at the damaged site [178]. The phosphorylation of H2AX at Ser 139 serves as a signal to recruit DNA repair proteins. These proteins are involved in either the HR or NHEJ pathway, along with checkpoint proteins that mediate cell-cycle arrest to allow for DNA repair [179,180]. In response to DNA damage, CDK2 activates checkpoint pathways, such as ATM, ATR, or the ATM/ATR-P53-P21 pathway, to initiate DNA repair [181]. In porcine embryos, DNA damage repair through the ATM-P53-P21-dependent cell-cycle checkpoint pathway can be initiated by CDK2 or, in the case of its inhibition, by CDK1, which is reported to compensate for CDK2 activity [56]. However, CDK2 also regulates apoptosis and the expression of genes involved in DSB repair, such as RAD51, MRE11a, PRKDC, and 53BP1, which are part of the HR and NHEJ pathways [56]. In pigs, as in other species, late-cleaving embryos show higher DNA damage than their early-cleaving counterparts [52,182,183]. The delayed cleavage is accompanied by the increased expression of HR and NHEJ DNA repair pathway genes and cell-cycle checkpoint genes, CHEK1 and CHEK2 [182]. It was suggested that the delayed cleavage is a response to DNA damage encountered during early embryo development. Though both HR and NHEJ pathways are activated in response to DSBs, the ATM-regulated HR pathway is the main mechanism of DNA damage repair in IVP-, SCNT-, or PA-produced porcine embryos [184].

### Post-Translational Modifications

Post-translational modifications (PTMs) play an important role in biological processes, including DDR. Their role is significant during transcriptional repression in mature oocytes and zygotes. During this period, PTMs are crucial in driving processes such as fertilization, initial cell division, and the transition from maternal to embryonic control. The importance of PTM in oocyte maturation, embryo development, DDR, and the clearance of maternal transcripts from the embryo has been reported in many species (reviewed in [185]). The most common PTMs include ubiquitination, SUMOylation, acetylation, methylation, Poly ADP-ribosylation (PARylation), and phosphorylation, among others. Ubiquitin proteasomal degradation is active during porcine oocyte meiosis and the initiation of mitosis in early embryos. The degradation of CCNB1 and phosphorylation of MAPK, which is required for the exit from M-II arrest, is reported to be driven by the ubiquitin-mediated degradation of these proteins in mice [186]. During early development of IVP embryos, selective autophagy is important for the recycling of degraded proteins of maternal origin to support the survival of mouse embryos [187]. In porcine embryos, expression of the enzyme, PARP1, involved in PARylation, is increased after fertilization and is related to the developmental competence of blastocysts. PARylation regulates pro-survival autophagy via the mTOR pathway by degrading ubiquitinated substrates and affects survival in porcine embryos [188,189]. As mentioned earlier, PTMs play an important role in the normal cell cycle and DNA damage response. Regarding this, the RNAseq analysis of 2-cell and 4-cell embryos revealed that, whereas ubiquitin conjugating (E2) enzymes involved in DNA repair or cell-cycle progression are expressed before zygotic genome activation, ubiquitin E3 ligases are expressed at higher levels in the 4-cell stage. These results point towards a stage-of-development-dependent role of ubiquitination in porcine embryos [12]. A stage-specific (oocyte 2- and 4-cell, as well as blastocyst) expression of Ubiquitins and SUMOs is involved in oocyte maturation and porcine embryo development, suggesting their role in early events of embryonic development [190]. This is evidence towards complex mechanisms that participate in safeguarding the integrity of the genome involving ubiquitination or SUMOylation PTMs. UV-induced DNA damage in blastocysts increases the expression of many genes, including RNF168 and RNF8, which are involved in the ubiquitin-mediated response to DSBs for DNA repair through HR [190]. Other ubiquitin ligases, such as DCAF13 and RNF114, participate in the normal regulation of early porcine embryo development [191]. The silencing of DCAF13 impairs EGA by decreasing the expression of KDM5B and KDM5C, consequently decreasing embryo development to the blastocyst stage. Moreover, both DCAF13 and RNF114 seem to be involved in DNA damage repair by regulating the expression of DNA recombinase RAD51 and phosphorylation of H2A.X.

## 7. Effect of In Vitro Culture Conditions on the Molecular Changes in Embryo

**Figure 3:**
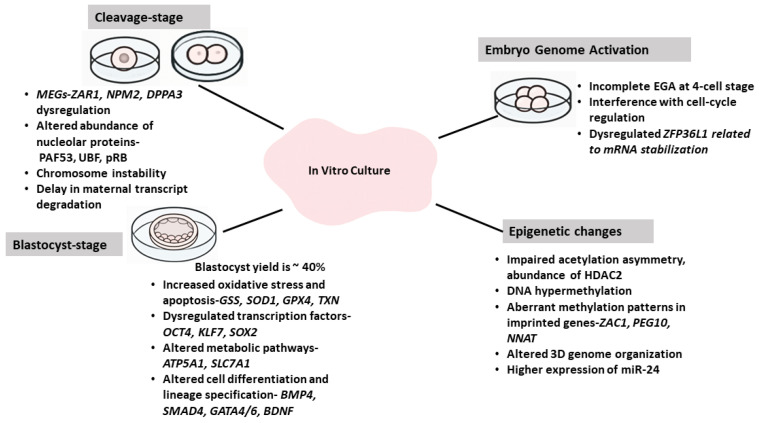
In vitro culture conditions induce dysregulations at various stages of development.

Although significant efforts have been made to improve in vitro embryo culture systems, resulting in overcoming the 4-cell developmental block in porcine embryos (reviewed in [192]), their efficiency remains unsatisfactory. The blastocyst yield in farm animals ranges at best to 40%. The porcine embryo represents an important agricultural and biomedical model for studying early mammalian development and improving reproductive biotechnologies. However, embryos produced in vitro have reduced developmental competence and pronounced transcriptional and epigenetic dysregulations as compared to their in vivo counterparts. Under natural conditions, early embryo development occurs within the oviduct and uterine lumen, which provides a tightly regulated biochemical and biophysical environment. In contrast, IVP embryos develop under artificial conditions that often compromise regular gene expression and epigenetic reprogramming [Figure 3]. Several chemical and physical factors, such as media formulation, gas atmosphere, temperature, pH, and osmolarity, impact embryo development during the culture (reviewed in [117,193,194,195,196,197]).

### 7.1. Effect of In Vitro Culture on Maternal Transcripts, Embryonic Genome Activation, and Early Development

The transition from maternal to embryonic control represents a critical developmental milestone, and any disturbance in transcript stability or utilization may lead to developmental arrest. Oocyte-specific mRNAs are selectively polyadenylated and translated to support cleavage divisions [198,199]. Perturbations in this balance, frequently observed under in vitro conditions, can result in premature or delayed transcript degradation and impaired EGA [52].

Maternal-effect genes, such as *ZAR1*, *NPM2*, and *DPPA3* (Stella/PGC7), are particularly sensitive to in vitro perturbations. Studies revealed that in vitro conditions affect *ZAR-1* expression as compared to in vivo development [200]. A decrease in *ZAR-1* mRNA level begins from the 4-cell stage in vivo, coinciding with EGA, whereas in vitro, the decline occurs at the 2-cell stage. The significant decrease in *ZAR-1* expression at the 2-cell stage of IVP embryos suggests premature utilization of this transcript, which may contribute to the reduced developmental competence of in vitro-cultured embryos. The supplementation of culture media with reproductive tract factors, such as EGF or IL-1β, modulates the expression of *ZAR1*, *NPM2*, and *DPPA3*, indicating that specific growth factors can partially restore physiological transcriptional profiles [201]. In addition to affecting maternal transcripts, in vitro-culture conditions alter the expression of nucleolar proteins, such as PAF53 and UBF, as well as cell-cycle regulator pRb. Disturbances in the expression of these genes can delay the EGA [202].

Numerous animal studies using high-fidelity RNA amplification techniques and microarrays analyzed the global transcriptome profile and revealed that an artificial environment modifies the expression of groups of genes and gene networks in the embryo at the transcriptomic level [12,13,14,135,203,204,205,206,207]. The comparison of porcine embryos before and after EGA shows that in vitro culture conditions significantly alter RNA profiles before EGA, suggesting that the artificial environment primarily affects maternal transcript utilization rather than zygotic transcription [12]. Before EGA, the IVP embryos showed an increased expression of EIF related to chromosome instability and genes associated with apoptosis, such as PDCD5, APAF1, and DIABLO. Molecular changes before EGA are presumed to originate primarily from oocytes due to their in vitro maturation conditions [12,14]. These findings underscore the importance of oocyte quality and its molecular content for subsequent embryonic viability. During maternal-to-zygotic transition and EGA, the maternal mRNA degradation differs between the IVP and in vivo produced embryos. This was suggested to be due to incomplete degradation of transcripts of phosphoprotein phosphatases, thus interfering with the cell cycle. The expression of genes related to stabilizing mRNAs, such as ZFP36L1 and ZFP36L2, is also dysregulated in IVP embryos due to the incomplete EGA at the 4-cell stage [14].

The morula stage of porcine IVP embryos showed reduced expression of the genes associated with response to oxidation stress and mitochondrial function [208], which was also observed in IVP bovine morulae [209]. At the blastocyst stage, some transcripts, such as *BMP4*, *KIT*, *GATA4/6*, *SMAD4*, *EZH2*, *ROCK1*, *FN1*, and *BDNF*, were among those altered in IVP embryos, potentially affecting their differentiation, lineage specification, and developmental competence [210]. The in vitro culture also resulted in an increase in many transcripts linked to antioxidant defense, such as *GSS*, *GPX4*, *SOD1*, *TXN*, *GSTA*, and apoptosis genes *TP53INP1*, *FAS*, *AIFM1*, and *BAX*, indicating that in vitro embryos experienced elevated oxidative stress and cell death [210].

Recently, the stress due to in vitro culture conditions was found to upregulate the p53 signaling pathway in IVP embryos as compared to their in vivo counterparts. Interestingly, the inhibition of the p53 pathway improved the development to the blastocyst stage with no effect on p53 target genes [207]. The study by Du et al. found that IVP and PA embryos showed a decreased expression of key TFs, such as OCT4, KLF7, SOX2, and SOX15, compared to in vivo-produced embryos [67]. Together, these data indicate that a transcriptional regulatory network composed of genes related to stress response, lineage specification, and key TFs could be fundamental to the in vitro development of porcine early embryos. The dysregulation of these processes can lead to reduced cell numbers and inner cell mass in IVP embryos.

Though IVP blastocysts with high developmental competence were similar to in vivo-produced embryos [211], multiple studies [13,135,166,211] highlighted perturbations in metabolic/stress pathways during the blastocyst stage. For example, the mitochondrial ATP synthase gene, *ATP5A1*, was more abundant in in vivo blastocysts than in IVP ones, suggesting that in vivo embryos had a higher mitochondrial activity. IVP embryos, on the other hand, showed upregulated amino acid transport and metabolic genes, including the arginine transporter *SLC7A1*, possibly reflecting compensatory stress or demand in less competent embryos [13,135]. The identification of *SLC7A1* as a transcript affected by in vitro culture prompted studies to evaluate arginine supplementation in porcine in vitro culture medium. Arginine supplementation not only promoted EGA but also led to the normalization of some of the transcriptomic differences, improving embryo developmental outcomes [71,166].

### 7.2. Effect of In Vitro Culture Conditions on Epigenetic Changes

Epigenetic regulation is particularly sensitive to the culture environment during the in vitro maturation of oocytes and in vitro culture of embryos [212,213]. Fertilization is followed by the massive epigenetic remodeling of the parental genomes, except for imprinted genes [214]. In vivo-matured porcine oocytes showed higher monospermy rates and were more competent at remodeling sperm chromatin in the male pronucleus. Global demethylation patterns in male pronuclei were significantly higher in zygotes obtained from in vivo-matured oocytes as compared to in vitro-matured and in vitro-fertilized zygotes [215]. Moreover, histone acetylation was asymmetric between male and female pronuclei, with male pronuclei being hyperacetylated relative to female pronuclei. This asymmetry was disturbed under in vitro conditions [215,216]. The abnormal abundance of histone deacetylase 2 (HDAC2) was implicated in disturbing acetylation asymmetry in porcine zygotes obtained after in vitro maturation of oocytes [215]. In vitro-produced porcine embryos had higher levels of DNA methylation compared to in vivo-produced embryos [213]. Blastocysts derived from in vitro fertilization exhibited altered DNA methylation, with incomplete or abnormal demethylation/remethylation dynamics and higher overall DNA methylation than in vivo embryos. These changes in methylation were developmental stage-dependent and lineage-specific (trophectoderm vs. inner cell mass) [213,217,218]. Whole-genome bisulfite sequencing of single porcine blastocysts showed locus-specific differences among in vivo and IVP embryos, including in regulatory regions of developmentally important genes. Genes involved in reprogramming, imprinting, and development were affected by culture conditions [212,213]. Aberrant methylation patterns in imprinted genes, such as ZAC1, PEG10, and NNAT, were observed under in vitro culture conditions [219]. The differences highlighted that the blastocyst environment (in vivo vs. in vitro) shaped early methylation patterns. The study by Canovas et al. [218] showed that adding reproductive fluids partly restored more physiologic methylation and transcriptomic patterns in IVP embryos. High-resolution Hi-C used to profile 3D chromatin architecture revealed that IVP porcine embryos exhibited an asynchronous establishment of higher-order chromatin structures (A/B compartment switches, TAD formation) as compared to uniparental embryos. The asynchrony correlates with altered transcriptional dynamics and suggests that in vitro fertilization perturbed the timing and the fidelity of 3D genome organization [111]. Though how three-dimensional architecture of chromatin differs between in vivo and IVP embryos is not known.

Studies investigating the effect of culture conditions on miRNA profiles in porcine early embryos are scarce. However, the expression of key miRNAs, such as miR-24, was expressed at significantly higher levels in IVP embryos as compared to their in vivo counterparts [220]. The increased expression of miR-24 in IVP embryos was predicted to target genes regulating inflammatory response, potassium ion transport, and responses to external stimuli, potentially leading to reduced embryo viability. While the exact roles of many miRNAs are still being investigated, the altered miRNA landscape in IVP embryos and other epigenetic changes suggest that epigenetic modifications induced by the in vitro environment negatively affected gene expression and developmental potential in pigs and other species [220,221].

## 8. Conclusions

These studies reviewed here highlight the importance of oocyte maturation and early embryonic environment to determine the timing and accuracy of epigenetic reprogramming and chromatin remodeling. During EGA, TFs operate within a metabolically sensitive and epigenetically remodeled chromatin landscape, potentially cooperating with other pioneer factors such as DUXA, LEUTX, and broader transcriptional machinery involving RNA polymerase, to effect the onset of the embryonic transcriptional program. Moreover, there are differences in the timing of maternal transcript degradation and the expression of key TFs and epigenetic modulators between in vivo and IVP embryos (IVF, PA, SCNT) that could be responsible for the poor outcomes in in vitro development. In vitro manipulations often introduce asynchronies and aberrations in methylation and 3D genome architecture; however, biomimetic approaches, such as inclusion of components of oviductal fluids and targeted modulation of chromatin regulators, hold promise in reducing in vitro culture-related epigenetic perturbations and improving developmental outcomes.

## Data Availability

No new data were created or analyzed in this study. Data sharing is not applicable to this article.
